# Is Obstructive Sleep Apnea Associated with Cardiovascular and All-Cause Mortality?

**DOI:** 10.1371/journal.pone.0069432

**Published:** 2013-07-25

**Authors:** Xiahui Ge, Fengfeng Han, Yanxi Huang, Yue Zhang, Tianyun Yang, Chong Bai, Xuejun Guo

**Affiliations:** 1 Department of Respiratory Medicine, Xinhua Hospital, Shanghai Jiao Tong University, Shanghai, China; 2 Department of Respiratory Medicine, Changhai Hospital of Second Military Medical University, Shanghai, P. R. China; University of Surrey, United Kingdom

## Abstract

**Background:**

Studies have reported inconsistent findings regarding the association between obstructive sleep apnea (OSA) and future risks of cardiovascular and all-cause mortality. We conducted a meta-analysis to investigate whether OSA is an independent predictor for future cardiovascular and all-cause mortality using prospective observational studies.

**Methods:**

Electronic literature databases (Medline and Embase) were searched for prospective observational studies published prior to December 2012. Only observational studies that assessed baseline OSA and future risk of cardiovascular and all-cause mortality were selected. Pooled hazard risk (HR) and corresponding 95% confidence intervals (CI) were calculated for categorical risk estimates. Subgroup analyses were based on the severity of OSA.

**Results:**

Six studies with 11932 patients were identified and analyzed, with 239 reporting cardiovascular mortality, and 1397 all-cause mortality. Pooled HR of all-cause mortality was 1.19 (95% CI, 1.00 to 1.41) for moderate OSA and 1.90 (95% CI, 1.29 to 2.81) for severe OSA. Pooled HR of cardiovascular mortality was 1.40 (95% CI, 0.77 to 2.53) for moderate OSA and 2.65 (95% CI, 1.82 to 3.85) for severe OSA. There were no differences in cardiovascular mortality in continuous positive airway pressure (CPAP) treatment compared with healthy subjects (HR 0.82; 95% CI, 0.50 to 1.33).

**Conclusions:**

Severe OSA is a strong independent predictor for future cardiovascular and all-cause mortality. CPAP treatment was associated with decrease cardiovascular mortality.

## Introduction

Obstructive sleep apnea (OSA) is characterized by repetitive episodes of complete or partial obstructions of the upper airway during sleep. Prevalence of OSA with an apnea-hypopnea index (AHI) exceeding 10–15 is 7–10% in the general adult population [1], and approximately 2–4% of the adult population between the ages of 30 and 60 years occurs excessive daytime somnolence [2]. Untreated OSA is associated with significant cardiovascular morbidity and mortality, debilitating daytime symptoms and increased risk of work and motor vehicle accidents.

OSA is highly prevalent in patients with hypertension, coronary artery disease, stroke, and atrial fibrillation [3], [4]. OSA has been reported to be associated with increased cardiovascular mortality [5], [6], [7], [8], [9] and all-cause mortality [6], [9], [10], [11], [12], [13], [14], and in particular with coexistence of OSA and cardiovascular disease [15], [16], [17], [18], [19]. However, many of these reports did not examine the contributing role of confounding factors [14], nor the relationship with the severity of OSA [7], [13]; conflicting results whether this association is independent of obesity and co-morbidities remain [6], [9], [12], [14].

To the best of our knowledge, no meta-analyses of such studies have been conducted on the association between OSA and future risk of cardiovascular and all-cause mortality. Given these reasons, a meta-analysis may help clarify this issue. The objective of the current meta-analysis was to quantitatively evaluate findings from prospective observational studies on OSA and future risk of cardiovascular and all-cause mortality, and determine whether OSA is an independent predictor of cardiovascular and all-cause mortality.

## Methods

### Search Strategy

We conducted a PubMed database and Embase search (up to December 2012) for studies assessing the association between OSA and future risk of cardiovascular and all-cause mortality. Papers could be published in English and/or Chinese. Potentially relevant studies included the word ‘mortality’, ‘death’ plus at least one of the following terms: sleep apnea, obstructive apnea, sleep-disordered breathing, obstructive sleep apnea, obstructive sleep hypopnea, sleep hypopnea syndrome, and upper airway obstruction. In addition, we also manually searched the reference lists to detect additional eligible studies.

### Study Selection

Studies satisfying the following criteria were included in the prospective observational meta-analysis: 1) adults who had been diagnosed with OSA, of any severity, confirmed by using a standardized polysomnography; and 2) providing adjusted hazard risk (HR) and the 95% confidence interval (CI) dealing with the risk of cardiovascular and all-cause mortality with varying degrees of OSA severity patients compared with without OSA. In addition, of the included studies, we also compared the patients with continuous positive airway pressure (CPAP) treatment OSA with untreated subjects. CPAP treatment was defined the start of treatment and the average cumulative adherence was 4 or more hours per day. Untreated CPAP was defined as no treatment prescribed or the patient declined to utilize treatment or could not tolerate the device or was persistently noncompliant (average use <4 hours/day).Studies were excluded if 1) the study design was a case-control study or retrospective design; 2) unadjusted HR was reported; and 3) not reporting results for moderate and/or severe OSA.

### Outcomes Measures and Data Extraction

Outcome measures included cardiovascular mortality (defined as death from stroke, heart failure, myocardial infarction or arrhythmia), and all-cause mortality. Death at the end of follow-up was obtained from the medical records, or from official death certificates.

AHI or the respiratory disturbance index (RDI) is the most commonly used to assess the severity of the OSA. According to the International Classification of Sleep Disorders, OSA is defined as AHI >15/h in an asymptomatic patient or AHI >5/h in a patient with excessive daytime sleepiness or combining symptoms and an RDI ≥5 or by an RDI ≥15 without symptoms [20]. A widely-used cutpoint at 5, 15 and 30 identified mild, moderate, and severe OSA, respectively.

Two reviewers (Xiahui Ge and Xuejun Guo) independently extracted the data from each trial. The HR and 95% CI were extracted. We used the fully adjusted HR for all of the included studies. We also extracted the following items from everyone study: author; year of publication; the location of study; methods used in diagnosing OSA; CPAP treatment; duration of follow-up; the sample size, gender, and the mean age or age range of participants; mortality events; and statistical adjustments for confounding factors. Where discrepancies were identified, reviewers resolved these by discussion.

### Quality Assessment

Quality assessment was performed with consideration for the following aspects followed the Meta-analysis of Observational Studies in Epidemiology guidelines [21]: 1) clear inclusion and exclusion criteria; 2) documentation of the loss to follow-up rate; 3) clear definition of outcome and outcome assessment; 4) sufficient duration of follow-up; 5) appropriate statistical analysis; and 6) important confounded and prognostic factors identified. All items had the following answer options: yes/no/too little information to answer the question.

### Statistical Analyses

The HR was used as the common measure estimate across studies. Data analyses used multivariate-adjusted HR and 95% CI. Before pooling the data, adjusted HR from each study was converted to their logHR to stabilize the variances and to normalize the distributions.

Homogeneity of HR across studies was assessed using the Cochrane Q statistic (p<0.10 was considered indicative of statistically significant heterogeneity) and I^2^ statistic (values of less than 40% as “heterogeneity might not be important” and of more than 75% as “considerable heterogeneity, based on the suggestion of the Cochrane Handbook for Systematic Review of Interventions) [22]. As there was substantial heterogeneity in the types of OSA diagnosis and follow-up duration between the different studies, a random effects model (Mantel-Haenszel heterogeneity) was used to calculate the pooled HR.

Potential publication bias was also assessed by both the Begg’s rank correlation test [23] and Egger linear regression test at p<0.10 [24]. Finally, sensitivity analysis was used to investigate the influence of a single study on the overall risk estimate, and was carried out by sequentially omitting one study at each turn with the metaninf algorithm in STATA. All analyses were performed using STATA version 12.0 statistical software (Stata Corp LP, College Station). P<0.05 was considered as statistically significant.

## Results

### Literature Search

**Figure 1:**
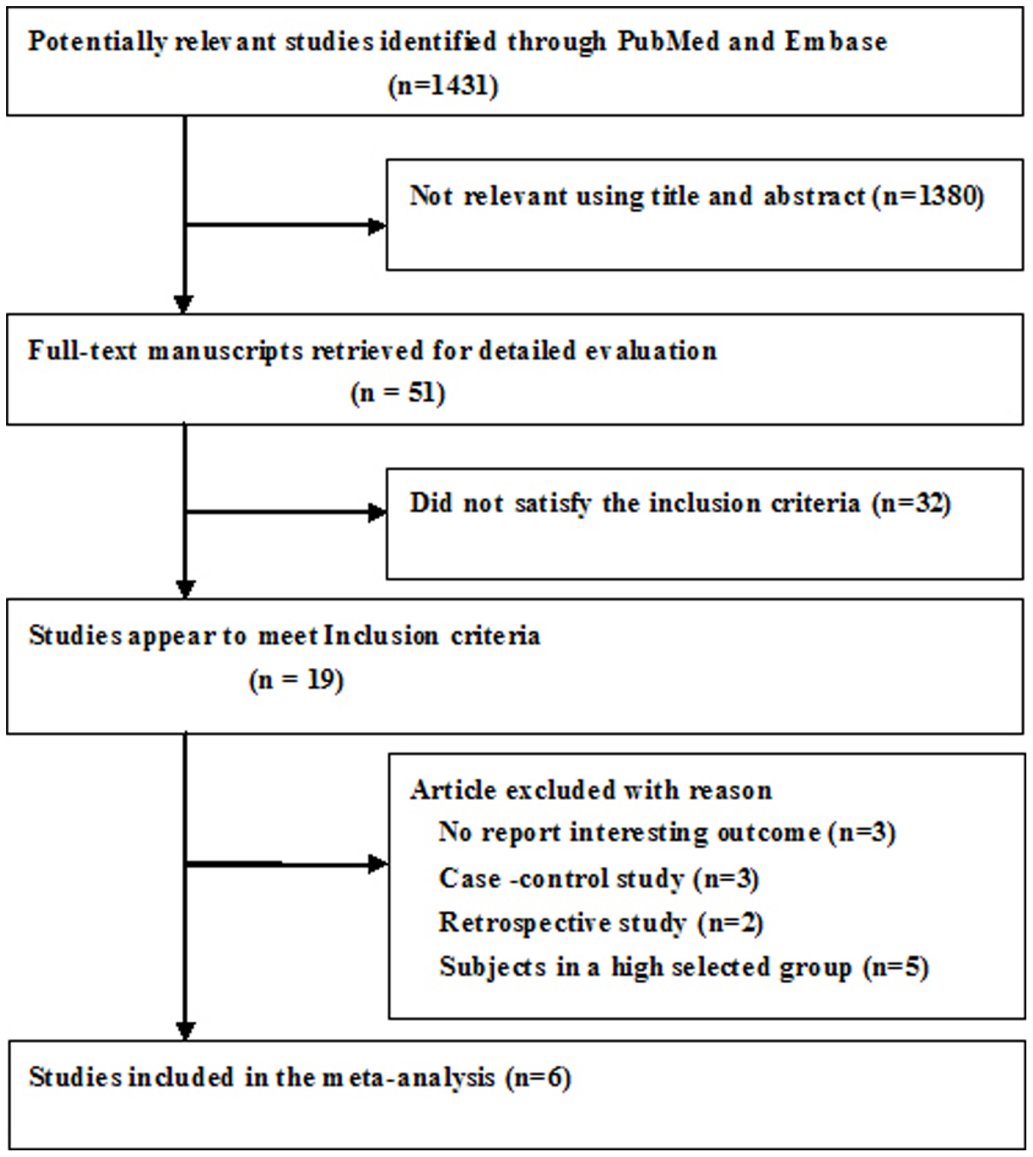
Flow chart of study selection process for meta-analysis.

Following the application of the search strategy, a total of 1431 potentially relevant citations were identified in our initial literature search. After screening the abstracts or titles, 1380 studies were excluded, mainly because they were reviews, case-control studies, or not relevant to our analysis. After reviewing the full texts, six studies [5], [6], [8], [9], [10], [11] met the inclusion criteria. A flow chart showing the study selection is presented in Figure 1.

### Baseline Characteristics and Quality Assessment

The characteristics of the included studies are listed in Table 1. Qualities of the included studies are listed in Table S1.

**Table 1:** Summary of clinical studies included in meta-analysis.

**Study/year**	**Country**	**Design**	**Subjects (%men)**	**OSA assessment/Patient Selection**	**Age/Mean (SD)**	**BMI/median/mean(SD)**	**Follow-up(Year)**	**Outcome assessment**	**Outcome/Events number/HR or OR (95% CI)**	**Adjustment for Covariates**
Marin et al 2005 [5]	Spain	Prospective study	1651(100)	AHI ≥30 (235)AHI 5 to <30 (403)AHI <5 (264)Snorers (377)AHI>30 treated(372)	50·3 (8·1)49·9 (7·2)49·6 (8·1)49·9 (9·1)49·9 (8·5)	27·5(4.·4)30·3 (4·2)29·8 (4·4)26·1 (3·6)30·7 (4·4)	10·1	National Death Index Death certificate	CVD death (75)1·15 (0·34 to 2·69) S2·87 (1·17 to 7·51) m+M1.05 (0.39 to 2.21) CPAP	Age, diagnostic group, CVD, DB, hypertension, lipid disorders, smoking status, alcohol use, SBP, DBP, blood glucose, TC, triglycerides, and use of antihypertensive, lipid-lowering, and antidiabetic drugs.
Young et al 2008 [6]	USA	Prospective study	1522 (55)	AHI ≥30(63)AHI 15 to <30(82)AHI 5 to <15(220)AHI <5(1157)	50 (9)50 (8)50 (8)47 (8)	37.233.331.527.6	18	Social Security Death Index, Wisconsin State Bureau of Health Information and Policy, Vital Records Section	CVD death (23)5.20 (1.4 to 19.2) S1.5 (0.3 to 7.3) M1.3 (0.4 to 4.1) mAll-cause(80)3.8 (1.6 to 9.0) S1.7(0.7 to 4.1) M1.4 (0.7 to 2.6) m	Age, sex, BMI, hypertension (or use of antihypertensive medication), CHD, CVD, self-reported diagnosis of DB, heart failure, MI, cardiac surgery, and stroke.
Punjabi et al2009 [11]	USA	Prospective cohort study	6294(46.7)	AHI ≥30(341)AHI 15 to <30(727)AHI 5 to <15(1797)AHI <5(3429)	64.6 (10.7)65.1 (10.5)64.8 (10.6)61.3 (11.1)	32.1 (6.1)30.7 (5.8)29.5 (5.3)27.0 (4.5)	8.2	Multiple concurrent approaches	All-cause(1047)1.46(1.14 to 1.86) S1.17 (0.97 to 1.42) M0.93 (0.80 to 1.08) m	Age, sex, race, smoking status, BMI, SBP, DSP, hypertension, diabetes, and CVD.
Marshall et al 2008 [10]	Australia	Prospective study	380 (73.2)	RDI ≥15(18)RDI 5 to <15(77)RDI <5(285)	55.1 (8.2)54.3 (7.2)52.6 (7.5)	34.3 (7.3)27.9 (4.1)26.2 (3.7)	13.4	National Death Index Death certificates	All-cause(33)6.24(2.01 to 19.39) M+S0.47 (0.17 to 1.29) m	Age, gender, BMI, mean arterial pressure, TC, HDL, DB, and medically diagnosed angina
Martinez-Garciaet al 2012 [9]	Spain	Prospective study	939 (36)	AHI ≥30(173)AHI 15 to <30(108)AHI <15(155)OSA treated (n = 503)	71.9(4.5)71.7 (5.2)70.9 (4.4)70.1 (4.2)	34.8 (6)33.6 (4.4)32.4 (5.1)35.1 (5.9)	5.8	Death certificate Medical records	CVD death (100)2.25 (1.41 to 3.61) S1.38 (0.73 to 2.64) M0.93 (0.46 to 1.89) CPAPAll-cause(190)1.99 (1.42 to 2.81) S1.17 (0.75 to 1.81) M	OSA group, age, gender, type of sleep study, sleep clinic, BMI, DB, smoke habit, ESS, cardiovascular events, dyslipidemia, and hypertension
Campos-Rodriguez et al 2012 [8]	Spain	Prospective cohort study	1116 (0)	AHI >30 (95)AHI of 10–29 (167)AHI <10 (278)AHI>30 treated(421)AHI10–29 treated(155)	64.2 (11.4)58.2 (12.0)52.1 (12.5)59.1 (11.1)58.3 (9.8)	37.9 (7.3)35.1 (5.9)33.1 (6.2)38.7 (7.5)37.7 (7.3)	3	Death certificate Medical record	CVD death (41)3.50 (1.23 to 9.98) S1.60 (0.52 to 4.90) m+M0.55(0.17 to 1.74) CPAP(>30)0.19(0.02 to 1.67) CPAP (10–29)	Age, BMI, hypertension, DB, study group, and previous cardiovascular events.

Abbreviations: BMI, body mass index; AHI, apnea-hypopnea index; RDI, respiratory disturbance index; HR, hazard risk; OR, odds ratio; SBP, systolic blood pressure; DBP, diastolic blood pressure; MI, myocardial infarction; DB, diabetes mellitus; CVD, cardiovascular disease; CRP, C-Reactive protein; TC, total cholesterol; HDL, high-density lipoprotein cho­lesterol; AF, atrial fibrillation; CHD, coronary heart disease; ESS, Epworth Sleepiness Scale; m, mild OAS;M, moderate OSA;S, severe OSA; SSDI, Social Security Death Index; CPAP, continuous positive airway pressure.

### All-Cause Mortality

**Figure 2:**
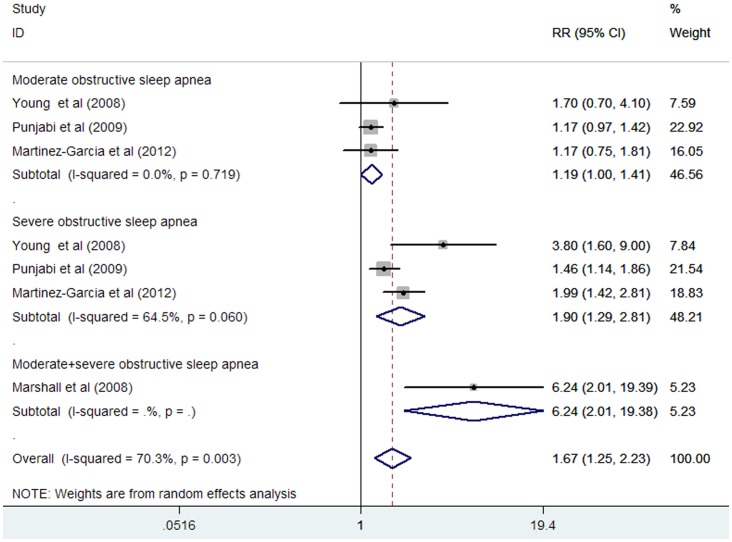
HR and 95% CI from the included studies of moderate and severe OSA with all-cause mortality comparing OSA to the control.

Three studies [6], [8], [11] reported on all-cause mortality for moderate and severe OSA, separately. One study reported the outcome of all-cause mortality for moderate to severe OSA [10]. The total number of participants included in this meta-analysis was 9165, with 1397 all-cause mortality. As shown in Figure 2, moderate to severe OSA was associated with an increase in all-cause mortality in a random effect model compared without OSA subjects (HR 1.67; 95% CI, 1.25 to 2.23; P = 0.001). Substantial heterogeneity was observed (I^2^ = 70.3%; P = 0.003). Evidence of publication bias for studies reporting adjusted HR of all-cause mortality for moderate to severe OSA was noted by the Begg’s rank correlation test (P = 0.051) and Egger’s linear regression test (P  = 0.036).

Subgroup analyses showed that moderate OSA was not associated with an increase in all-cause mortality compared without OSA subject (HR 1.19; 95% CI, 1.00 to 1.41;P = 0.051). Substantial heterogeneity was not observed (I^2^ = 0%; p = 0.867). Severe OSA was associated with an increase in all-cause mortality compared without OSA subjects (HR 1.90; 95% CI, 1.29 to 2.81; P = 0.001). Substantial heterogeneity was observed (I^2^ = 64.5%; P = 0.060).

### Cardiovascular Mortality

**Figure 3:**
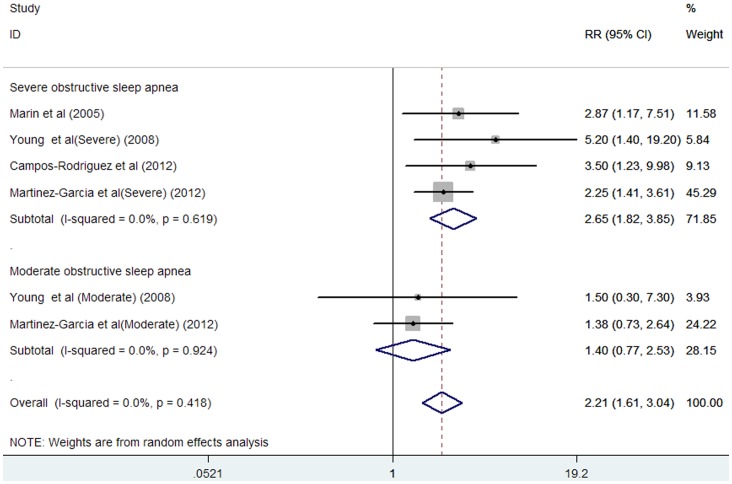
HR and 95% CI from the included studies of moderate and severe OSA with cardiovascular mortality comparing OSA to the control.

Four studies [5], [6], [8], [9] reported on cardiovascular mortality for moderate to severe OSA. The total number of participants included in this meta-analysis was 5228, with 239 reporting cardiovascular mortality. As shown in Figure 3, moderate to severe OSA was associated with an increase in cardiovascular mortality in a fixed-effect model compared without OSA subjects (HR 2.21; 95% CI, 1.61 to 3.04; P = 0.000). Substantial heterogeneity was not observed (I^2^ = 0%; P = 0.418). There was no evidence of publication bias for studies reporting adjusted HR of cardiovascular mortality, as suggested by the Begg’s rank correlation test (P = 0.452) and Egger’s linear regression test (P = 0.448).

Subgroup analyses showed that moderate OSA was associated with an increase in cardiovascular mortality compared to those without OSA (HR 1.40; 95% CI, 0.77 to 2.53; P  = 0.273). Substantial heterogeneity was not observed (I^2^ = 0%; P = 0.924).Severe OSA was associated with an increase in all-cause mortality compared to subjects without OSA (HR 2.65; 95% CI, 1.82 to 3.85; P = 0.000). Substantial heterogeneity was not observed (I^2^ = 0%; P = 0.619).

### Effects of CPAP Treatment on Cardiovascular Mortality

**Figure 4:**
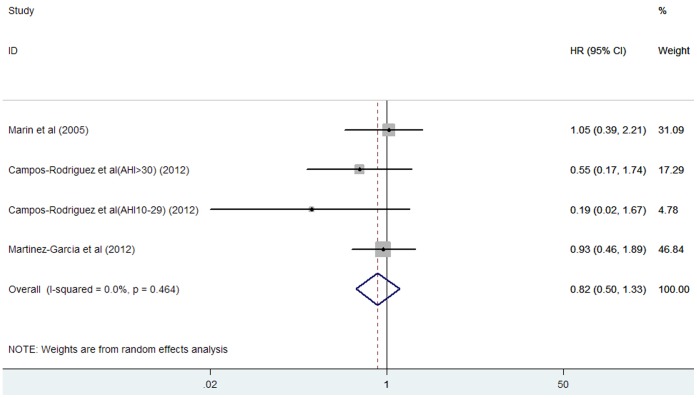
HR and 95% CI from the included studies of continuous positive airway pressure treatment with cardiovascular mortality comparing OSA to the control.

Three studies [5], [8], [9] reported on cardiovascular mortality for CPAP treatment. The total number of participants included in this meta-analysis was 1451, with 62 reporting cardiovascular mortality. As shown in Figure 4, there were no differences in cardiovascular mortality in CPAP treatment group compared with no OSA subjects (HR 0.82; 95% CI, 0.50 to 1.33; P = 0.414). Substantial heterogeneity was not observed (I^2^ = 0%; P = 0.464). There was no evidence of publication bias for the studies reporting adjusted HR of CPAP treatment on cardiovascular mortality, as suggested by the Begg’s rank correlation test (P = 0.734) and Egger’s linear regression test (P = 0.335).

### Sensitivity Analyses

Sensitivity analyses were performed based on all-cause and cardiovascular mortality. In the metainf analysis, there was little influence in the quantitative pooled measure of HR or 95% CI when omission of anyone studies as shown in Table S2 (all-cause mortality) and S3 (cardiovascular mortality).

## Discussion

The findings of the current meta-analysis provided evidence that severe OSA is a strong independent predictor for future cardiovascular and all-cause mortality. Subjects with severe OSA increased 67% risk of all-cause mortality and 265% risk of cardiovascular mortality. In addition, CPAP treatment was associated with decrease cardiovascular mortality.

OSA affects both sexes, but a two to threefold higher prevalence in men than women [1], [25]; the difference might be related to the differences in pharyngeal collapsibility and central respiratory drive [26]. In the current included studies, women with severe OSA increased risk of cardiovascular mortality (HR 3.50) [8]; in contrast, men with severe OSA were at a lower risk (HR 2.87) [5].As for all-cause mortality, OSA was associated with increased mortality in male, but not in female subjects [11]. However, due to the limited number of studies, we were unable to conduct subgroup analyses on gender. Therefore, whether men or women with severe OSA had more risk of mortality is still unclear.

The association between age and OSA is complex. OSA subjects had a higher mortality rate than subjects of the same age in the general population [27], [28], and appeared to have more excess mortality in patients younger than 50 years of age [28], [29].In the Sleep Heart Health Study, OSA was not associated with increased mortality in subjects over 70 years of age [11]. Another recently published study further demonstrated that OSA was not associated with mortality in persons >75 years old [14].OSA in the elderly may not be associated with the same increase in mortality than in middle-aged patients [30].In the current meta-analysis, the age of subjects in the included studies varied in these studies, and it might influence the findings of the meta-analysis. Hence, the generalizability of these findings is limited, as for the population studied varied with respect to age, sex, and obesity.

A few studies that did not meet the inclusion criteria for the meta-analysis also found severe OSA was associated with future cardiovascular and all-cause mortality. In a prospective study which recruited 147 hypertensive patients, the adjusted HR for mortality with severe OSA was 9.20 (1.177 to 72.00) [15]. In a total of 1022 consecutive patients, severe OSA was associated with the development of stroke or death (HR 1.97; 95% CI, 1.12 to 3.48) [16].In a retrospective cohort study of 281 consecutive OSA patients with a history of myocardial injury or with known existing ischemic heart disease, the adjusted HR for mortality with severe OSA was 1.72 (95% CI, 1.01 to 2.91) [17]. A recently published well-designed meta-analyses suggested that OSA was associated with an increased rate of stroke and cardiovascular mortality [31].However, these studies are mostly based on clinical samples rather than general population cohorts.

Exact mechanisms linking OSA with cardiovascular and all-cause mortality are not fully elucidated; however, several possible explanations are as follows: sympathetic activation, inflammation, oxidative stress, and endothelial dysfunction. All consequences of OSA are directly linked to intermittent hypoxia in the pathogenesis of cardiovascular disease in patients with OSA [32], [33]. An important issue is whether treatment of severe OSA with CPAP reduces mortality rates. CPAP is the mainstay of therapy for moderate to severe OSA. The current meta-analysis suggested that CPAP treatment was not associated with decrease cardiovascular mortality (HR 0.82; 95% CI, 0.50 to 1.33) compared with healthy subjects. A well–designed meta-analysis also demonstrated that CPAP was an effective treatment for OSA compared with conservative/usual care and placebo in populations with moderate to severe daytime sleepiness [34].

There are several potential limitations of this study. First, we only included studies published in English, and some relevant studies might be not included in the meta-analysis. Second, a major limitation was the possibility of uncontrolled confounding, and the individual studies did not adjust for potential risk factors in a consistent way. There are several known risk factors for OSA such as obesity, age, cigarette smoking, and craniofacial abnormalities. OSA often coexists with cardiovascular diseases that are often associated with a high mortality rate and that could obscure the influence of OSA on mortality. The lack of adjustment for these confounding factors might have resulted in a slight overestimation of the HR. Third, another limitation of the current study is that the severity of OSA at the initial diagnosis did not reflect the severity of the syndrome at the end. However, previous studies on variability on sleep and breathing patterns have shown that one night of recording provides a reasonably accurate estimate of OSA severity [35]. Fourth, AHI does not account for the severity of the hypoxemia induced by the apneas and hypopnea [36]. Fifth, subgroup analyses will further explain the source of heterogeneity. However, due to the limited studies included, we could not conduct further deepen our analyses of the data, such as by gender, age and obesity. In addition, the length of follow-up in studies (ranges from 3 to 18 years) is an additional limitation. It is difficult to determine beyond the duration of the follow-up studies in the meta-analysis with respect to long-term impact on cardiovascular and all-cause mortality.

In conclusion, this meta-analysis provides evidence that severe OSA is an independent predictor for future cardiovascular and all-cause mortality. CPAP treatment was associated with decrease cardiovascular mortality in OSA patients. More well-designed studies are needed to confirm how age and gender in OSA are potential predictors of cardiovascular and all-cause mortality.

## Supporting Information

Table S1 Quality assessment of studies included in the meta-analysis.
(DOC)

Table S2 HR and 95% CI by omitting each study from the eligible studies of all-cause mortality.
(DOC)

Table S3 HR and 95% CI by omitting each study from the eligible studies of cardiovascular mortality.
(DOC)
