# Correction: Is Obstructive Sleep Apnea Associated with Cardiovascular and All-Cause Mortality?

**DOI:** 10.1371/journal.pone.0095953

**Published:** 2014-04-17

**Authors:** The PLOS ONE Staff

There are errors in the “Methods,” “Results,” and “Discussion” section as listed below:

In the “Methods” section, under the sub-heading “Study Selection,” the second sentence is incorrect. The correct sentence for this section is: “In addition, of the included studies, we also compared the patients with continuous positive airway pressure(CPAP) treatment OSA with healthy control group."

In the “Results” section, under the sub-heading “Cardiovascular Mortality,” the second to last sentence is incorrect. The correct sentence for this section is: “Severe OSA was associated with an increase in cardiovascular mortality compared to subjects without OSA (HR 2.65; 95% CI, 1.82 to 3.85; P =  0.000).”

In the “Results” section, under the sub-heading “Effects of CPAP Treatment on Cardiovascular Mortality,” the second sentence is incorrect. The correct sentence for this section is: “As shown in Figure 4, there were no statistical differences in cardiovascular mortality in CPAP treatment group compared with without OSA subjects (HR 0.82; 95% CI, 0.50 to 1.33; P  =  0.414).”

In the “Discussion” section, the second to last sentence of the fourth paragraph is incorrect. The correct sentence for this section is: “The current meta-analysis suggested that there were no statistical differences in cardiovascular mortality between OSA patients who underwent CPAP treatment and healthy subjects (HR 0.82; 95% CI, 0.50 to 1.33).”
