# Supplementary material for: Is Obstructive Sleep Apnea Associated with Cardiovascular and All-Cause Mortality?
Source: PLoS One. 2013 Jul 25;8(7):e69432. doi: 10.1371/journal.pone.0069432 (PMC3723897; doi:10.1371/journal.pone.0069432)
Supplement: Table S1 — Quality assessment of studies included in the meta-analysis. (DOC) [file pone.0069432.s001.doc]

~~Supplement Table S 1 - Quality assessment of studies included in the meta-analysis.~~

| Study | Clear inclusion and exclusion criteria | Document the loss to follow up rate | Clear definition of outcome | Sufficient duration follow-up | Control of confounding | Appropriate statistics |
| --- | --- | --- | --- | --- | --- | --- |
| Marin et al 2005[5] | Yes | Yes | Yes | Yes | Yes | Yes |
| Young et al 2008[6] | Yes | Yes | Yes | Yes | Yes | Yes |
| Punjabi et al 2009[11] | Yes | No | Yes | No | Yes | Yes |
| Marshall et al 2008[10] | Yes | No | Yes | Yes | Yes | Yes |
| Martinez-Garcia et al 2012[9] | Yes | Yes | Yes | No | Yes | Yes |
| Hudgel et al 2012[12] | Yes | No | Yes | No | Yes | Yes |
| Campos-Rodriguez et al 2012[8] | Yes | Yes | Yes | No | Yes | Yes |
