# Supplementary material for: Is Obstructive Sleep Apnea Associated with Cardiovascular and All-Cause Mortality?
Source: PLoS One. 2013 Jul 25;8(7):e69432. doi: 10.1371/journal.pone.0069432 (PMC3723897; doi:10.1371/journal.pone.0069432)
Supplement: Table S2 — HR and 95% CI by omitting each study from the eligible studies of all-cause mortality. (DOC) [file pone.0069432.s002.doc]

**Supplement Table-S2** HR and 95% CI by omitting each study from the eligible studies of all-cause mortality.

| Study omitted | HR | 95% CI | |
| --- | --- | --- | --- |
| Marin | 2.1381397 | .58715147 | 7.7861362 |
| Young (Moderate) | 2.2477256 | .6505373 | 7.7663042 |
| Young(Severe) | 2.0981026 | .59968549 | 7.3405721 |
| Campos-Rodriguez | 2.1125971 | .59039293 | 7.5594852 |
| Martinez-Garcia(Severe) | 2.1815416 | .42188273 | 11.280679 |
| Martinez-Garcia(Moderate) | 2.5725671 | .63687902 | 10.391458 |
| Combined | 2.2122867 | .65622895 | 7.4580868 |
