# Supplementary material for: Is Obstructive Sleep Apnea Associated with Cardiovascular and All-Cause Mortality?
Source: PLoS One. 2013 Jul 25;8(7):e69432. doi: 10.1371/journal.pone.0069432 (PMC3723897; doi:10.1371/journal.pone.0069432)
Supplement: Table S3 — HR and 95% CI by omitting each study from the eligible studies of cardiovascular mortality. (DOC) [file pone.0069432.s003.doc]

**Supplement Table-S3** HR and 95% CI by omitting each study from the eligible studies of cardiovascular mortality.

| Study omitted | HR | 95%CI | |
| --- | --- | --- | --- |
| Young(Moderate) | 1.4082992 | .85796645 | 2.3116366 |
| Young (Severe) | 1.3829597 | .84231822 | 2.2706116 |
| Punjabi(Moderate) | 1.6493991 | .85209386 | 3.1927438 |
| Punjabi (Severe) | 1.3969732 | .78630471 | 2.4819057 |
| Martinez-Garcia(Moderate) | 1.4385906 | .8618297 | 2.4013362 |
| Martinez-Garcia (Severe) | 1.3373658 | .78815554 | 2.269282 |
| Marshall et al | 1.3871229 | .84680416 | 2.2722017 |
| Combined | 1.4138402 | .86582936 | 2.3087044 |
